# Attitudes, practices and information needs regarding novel influenza A (H7N9) among employees of food production and operation in Guangzhou, Southern China: a cross-sectional study

**DOI:** 10.1186/1471-2334-14-4

**Published:** 2014-01-02

**Authors:** Tiegang Li, Jing Feng, Pengzhe Qing, Xiaomei Fan, Weisi Liu, MeiXia Li, Ming Wang

**Affiliations:** 1 Guangzhou Center for Disease Control and Prevention, No 1, Qide Rd, Jiahe, Baiyun District, Guangzhou, Guangdong Province 510440, China; 2 Basic Medical College, Inner Mongolia Medical University, Hohhot 010059, China

**Keywords:** Attitudes, Health education, Influenza A (H7N9), Information needs, Practices

## Abstract

**Background:**

As of 30 May 2013, 132 human infections with avian influenza A (H7N9) had been reported in 10 Chinese cities. On 17 May 2013, because a chicken infection with H7 subtype avian influenza virus was detected in Guanzhou, Guangzhou became the 11th city to conduct emergency response operations. The goal of this study was to identify attitudes, practices and information needs among employees of food production and operation in Guangzhou.

**Methods:**

A cross-sectional survey of face-to-face interviews was used during 17–24 June 2013. All adults seeking health examination in Guangzhou Center for Disease Control and Prevention who had lived in Guangzhou for at least 3 months, were engaged in food production and operation, and agreed to participate were interviewed.

**Results:**

Of 1,450 participants, 69.72% worried about being infected with the A/H7N9 and 74.41% stated that they had searched for information about A/H7N9. The internet (76.92%), television (67.56%), and newspapers (56.26%) were the main methods of obtaining information; the use of these methods differed significantly by various demographic variables (P < 0.05). More than one-fifth of participants complained that the information was not timely enough (20.28%) and was intentionally concealed by the government (20.76%). Nearly one-third (32.35%) did not believe that the government could control the A/H7N9 epidemic. Most participants (80.76%) reported washing hands more frequently than before, while over one-third (37.17%) stated no longer buying poultry. A total of 84.00% indicated a willingness to receive an A/H7N9 vaccine, and the primary reason for not being willing was concern about safety (58.19%). A history of influenza vaccination and worry about being infected with the A/H7N9 were significantly associated with intention to receive an A/H7N9 vaccine (P < 0.05).

**Conclusions:**

Our findings provide insight into the attitudes and practices of employees of food production and operation 3 months after the first human A/H7N9 case reported in China, and 1 month after infected chickens were identified in Guangzhou. Distrust in the health department should be addressed, and more effort should be made to improve compliance of proper preventive measures to reduce panic among the public. The information needs should be taken into account in the next step of health education.

## Background

In early 2013, a novel strain of avian influenza A (H7N9) virus was detected in humans in Shanghai, Eastern China. The virus had never been reported in humans, and the World Health Organization is taking this novel A/H7N9 seriously. Most H7N9 patients have presented with respiratory tract infection with progression to severe pneumonia and breathing difficulties [1]. As of 30 May, a total of 132 human infections had been reported in 10 Chinese cities [2], of whom 37 died [3]. This yields a case fatality rate of 23.36%, which is substantially higher than seasonal influenza viruses, pandemic 2009 A/H1N1 virus [2], and other subgroups of H7 influenza A viruses (subtypes H7N2, H7N3, and H7N7) [4] in China. Although no person-to-person transmission or epidemiologic link between any of the cases has been identified, infection seemed to have involved contact with infected poultry [5]. The viral isolates from some patients were very similar to those from epidemiologically linked market chickens [5]. Furthermore, detection of more than 100 cases in 3 months compared with roughly 600 human cases of avian influenza A/H5N1 infections in a decade suggests that H7N9 is already more transmissible from poultry to humans than H5N1 [6].

**Figure 1:**
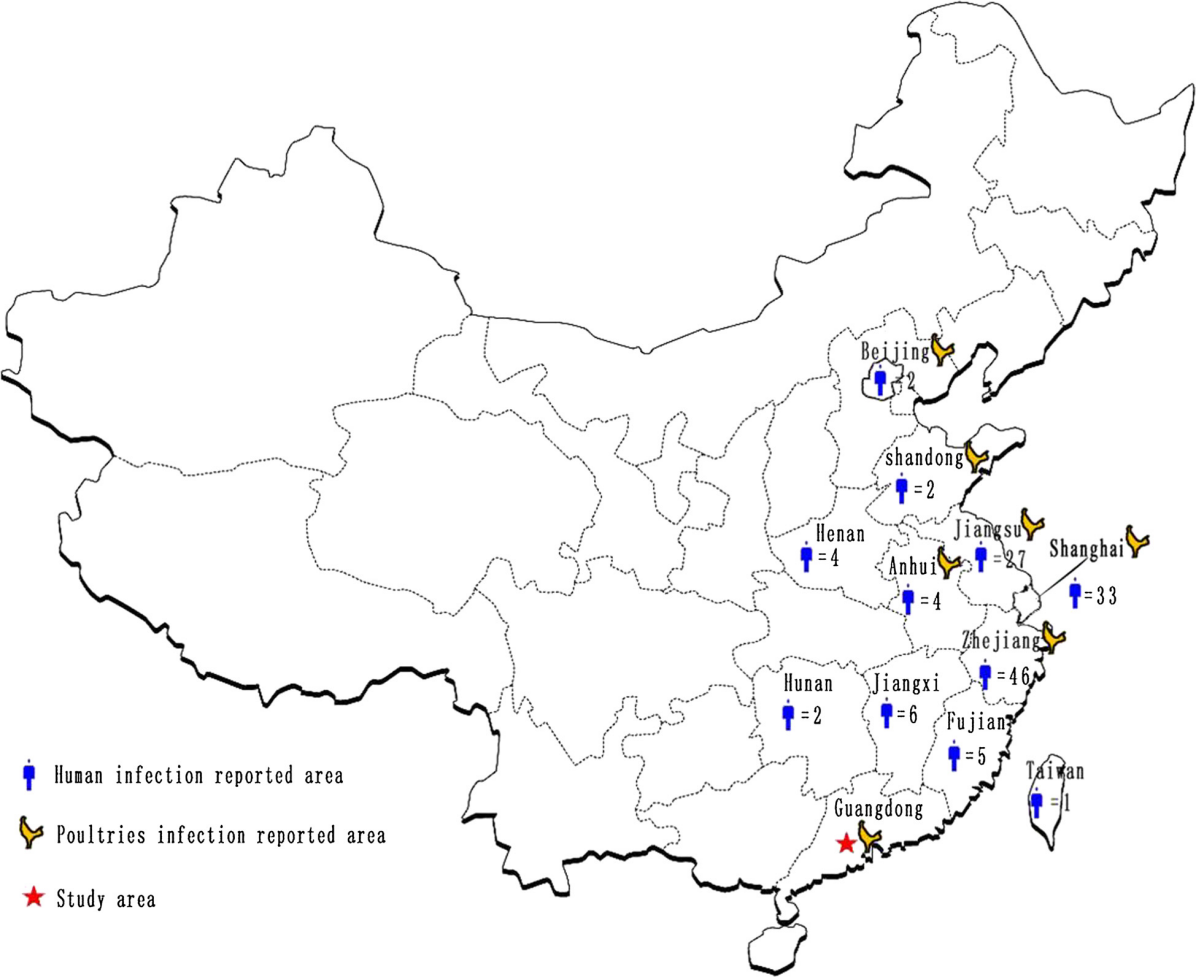
Areas in China where human and poultry H7N9 infections have been reported.

As the largest trading city of southern China, Guangzhou had a large burden of both Severe Acute Respiratory Syndromes(SARS) in 2003 and pandemic influenza A (H1N1) (pH1N1) in 2009 [7]. Although no human infections with the avian influenza A (H7N9) were reported in Guangzhou, human cases have been identified in Jiangxi, Hunan, and Fujian. These three provinces all border Guangzhou, and have frequent population movement and agricultural trade with Guangzhou (Figure 1). On 17 May 2013, the Guangzhou agriculture department announced that chicken samples from a poultry market tested positive for H7 subtype avian influenza virus. This led to Guangzhou being the 11th city in China to conduct emergency response operations related to novel influenza A (H7N9).

Public cooperation in complying with infection control measures is required to minimize the spread of infectious diseases. Previous studies have demonstrated the positive correlation between a willingness to adhere to the recommendations around infection control practices and perceived infectiousness and severity of the disease [8-10], perceptions about the effectiveness of control measures [11], and trust in the information being provided by national and international public health authorities [12]. Therefore, learning more about the concerns, attitudes, and behaviors of the public during an infectious disease epidemic is crucial to improve communication efforts by public health officials [13]. Because a considerable number of H7N9 patients engaged in food-related work before symptom onset (including chef, food transporter, poultry seller and slaughter), we conducted face-to-face interviews on attitudes, practices and information needs among employees of food production and operation in Guangzhou, with an effort to assess the preparedness and response of the public, and to facilitate the development of effective prevention strategies against H7N9 infection.

## Methods

### Study area

Guangzhou is 7,434 square kilometers in size, situated at 22°26′N – 23°56′N and 112°57′E – 114°3′E. As of the 2010 census, the city had over 7.9 million registered inhabitants and a floating population (such as migrant workers) of 4.8 million. It is the largest trading city in southern China, and is about 120 km north-northwest of Hong Kong and north-northeast of Macau (Figure 1).

### Participants and data collection

In China, according to the Public Places Health Management Regulations and Implementing Rules promulgated by the Chinese government, employees of food production and operation must accept a health examination every year, and it is illegal to engage in food-related work without a valid health certificate. There are 13 health examination centers available to the public in Guangzhou, and the centrally-located Guangzhou Center for Disease Control and Prevention (CDC) Health examination center is the largest. It is typically the first choice for employees, because people with a health certificate from Guangzhou CDC can legally work in all 12 districts of the city.

In this study, subjects were recruited by convenience sampling from the Guangzhou CDC health examination hall. All adults seeking health examination between 17–24 June 2013, who had lived in Guangzhou for at least 3 months, engaged in food production and operation, and agreed to participate in the investigation were interviewed face-to-face to complete a three-page questionnaire. Interviewers consisted of epidemiologists and dialect interpreters, and spent an average of 3 hours at a randomly chosen time of day to recruit participants. Each interview lasted about 20 minutes. All interviewers attended a 3-hour pre-training before conducting interviews. Because some questions in this study were about the government’s work, the responses might be untruthful if participants knew that this investigation was conducted by Guangzhou CDC; therefore, we masked our occupation when explaining the nature of this study to interviewees.

Pilot surveys were conducted prior to the study, to confirm that participants could understand the survey questions and to ensure the validity of the questionnaire content. Using the results of this pilot study, the survey questionnaire was amended to create a final version (Additional file 1). All questions were either closed-ended or multiple-choice.

### Attitudes

Nine questions were used to assess attitudes toward A/H7N9. One item was “do you worry about getting H7N9?”, and response options for this ranged from 1 = “absolutely not” to 6 = “very worried”. Six items related to “what do you think about the H7N9 information published by the government?”. Each of these six items was phrased as a statement, with response options “agree” and “disagree”. One item was related to “satisfaction evaluation on measures taken by the government”, and response options for this ranged from 1 =“very dissatisfied” to 5 =“very satisfied”. One item was “do you believe that government can control the H7N9 epidemic?”, and response options for this were “yes”, “hard to say”, and “no”.

### Practices

Participants were asked 10 questions about their recent practices. One question was “did you recently search for information about H7N9?” with response options of “yes” and “no”. If the response was “yes”, a supplementary question of “what methods or ways did you use?” was asked. Each method/way was phrased as a statement, and response options were “yes” and “no”. Seven items related to “preventive measures that have been taken after the emergence of H7N9”; all were phrased as “over the past months, I have … because of H7N9”, and the response options were “yes” and “no”. Two items related to vaccines. Participants were asked “have you received an influenza vaccine in the past three years?”, with response options of “yes” and “no”. Participants were also asked “if the H7N9 vaccine is available, would you like to receive it”. If the response was “no”, we asked “why?” as the supplementary question; there were three response options to this supplementary question.

### Information needs

Participants were asked to report any information needs or concerns if large numbers of human infections with H7N9 cases emerge or a H7N9 pandemic occurs in Guangzhou. There were 12 items for participants to respond with “yes” or “no”: Four related to the epidemic situation, one related to vaccination, three related to preventive measures, three related to drug treatment, and one related to poultry safety.

### Demographic variables

Demographic variables consisted of sex, age, household income, educational level, marital status, birthplace, living area, and length of time live in Guangzhou.

### Data analysis

Epi Info™ 7.1.0.6, a free statistical software package produced by the U.S. Centers for Disease Control and Prevention, was used for data entry, cleaning, and initial analysis. Descriptive statistics, such as percentages, means, and 95% confidence intervals, were calculated. A χ2 test and/or Fisher’s exact test were used to compare proportions of different groups. Multivariate logistic regression analyses were used to clarify the relationship between different methods of obtaining H7N9 information and demographic variables, and to identify the significant independent predictors of acceptance of a H7N9 vaccine, by calculating odds ratios (OR) after controlling for gender, age, and other demographics. These regression analyses were conducted using SPSS version 17.0 (SPSS Inc. 2008). P < 0.05 was considered statistically significant for all analyses.

### Ethical considerations

This study was approved by the Guangzhou Center for Disease Control and Prevention.

## Results

### Participant demographics

Of 1,542 subjects approached, 1,450 participants accepted and successfully completed the interview, yielding a response rate of 94.03%. The age ranged from 18 to 60 years old (mean: 23.91 years) (Table 1). The proportions of participants under age 25, 25–40, and over age 40 were 74.21%, 21.52%, and 4.28%, respectively. There were more females (N = 986, 68.00%) than males (N = 464, 32.00%). The majority of participants (60.90%) lived downtown, and more than half (56.07%) have lived in Guangzhou for more than 3 years. Nearly all (99.17%) participants had a middle school diploma or higher. Half of the participants had an annual household income per capita less than $6,000 (Table 1).

**Table 1:** Demographic characteristics of study participants in Guangzhou, 17–24 June 2013 (N = 1,450)

**Characteristics**	**No.**	**%**	**95% CI Lower**	**95% CI Upper**
**Age group(years)**				
<25	1076	74.21	71.86	76.43
25–40	312	21.52	19.45	23.74
>40	62	4.28	3.32	5.48
**sex**				
Male	464	32.00	29.62	34.48
Female	986	68.00	65.52	70.38
**Annual household income per capita**				
Less than $2,000	151	10.41	8.91	12.13
$2,001–$6,000	577	39.79	37.27	42.37
$6,001–$10,000	476	32.83	30.42	35.32
$10,001–$20,000	180	12.41	10.78	14.25
>$20,000	66	4.55	3.56	5.79
**Residence time in Guangzhou**				
Less than 1 year	348	24.00	21.84	26.30
1–3 years	279	19.24	17.26	21.39
**≥**3 years	813	56.07	53.47	58.64
Refused	10	0.69	0.35	1.31
**Highest education**				
Non-educated	5	0.34	0.13	0.85
Primary school graduate	7	0.48	0.21	1.04
Middle school graduate	189	13.03	11.37	14.90
High school graduate	560	38.62	36.11	41.19
College: 1–3 years technical school training	283	19.52	17.53	21.67
College: 4 years or more (college graduate)	400	27.59	25.31	29.98
Master or doctor degree graduate	6	0.41	0.17	0.95
**Living area**				
Suburban or rural	558	38.48	35.98	41.05
Downtown	883	60.90	58.32	63.41
Refused	9	0.62	0.30	1.22

CI = Confidence Intervals.

### Attitudes

At the time of this study, which took place 3 months after the first A/H7N9 human infection was reported and 1 month after the government announced that a chicken in Guangzhou market was infected, the majority of participants (69.72%) worried about being infected with the A/H7N9 (Table 2). When participants were asked about their opinions on H7N9 information published by the government, 73.03% reported “accurate and transparent”. However, more than a fifth (20.28%) reported “not timely enough”, and 20.76% thought “some information was intentionally concealed by the government”. With regard to satisfaction on the measures taken by the government, 93.17% of participants chose “satisfied”, “more satisfied”, or “very satisfied”. A total of 64.48% of participants believed that the government could control the H7N9 epidemic. However, nearly one-third (32.35%) stated “hard to say” or “no” (Table 2).

**Table 2:** Attitudes towards A(H7N9) influenza among employees of food production and operation in Guangzhou, 17–24 June 2013

**Items**	**No.**	**%**	**95% CI lower**	**95% CI upper**
**Did you worry about getting H7N9 virus?**				
Very worry	254	17.52	15.61	19.59
More worry	296	20.41	18.39	22.60
Worry	461	31.79	29.41	34.27
Not to matter	48	3.31	2.48	4.40
Not worry	391	26.97	24.71	29.34
Absolutely not worry	0	0.00	0.00	0.00
**How do you think about the H7N9 information published by government?**				
Accurate and transparent	1059	73.03	70.66	75.29
Not timely enough	294	20.28	18.25	22.46
Difficult to understand, more puzzling	205	14.14	12.41	16.06
Publics have limited access to get correct and authority information	235	16.21	14.37	18.23
The information was intentionally concealed by Government	301	20.76	18.72	22.96
The severity of epidemic was deliberately exaggerated by government, causing panic	104	7.17	5.92	8.65
**How about your personal satisfaction evaluation on the measures taken by government?**				
Very dissatisfied	94	6.48	5.30	7.91
Dissatisfied	5	0.34	0.13	0.85
Satisfied	248	17.10	15.22	19.16
More satisfied	546	37.66	35.16	40.21
Very satisfied	557	38.41	35.91	40.98
**Do you believe that government can control the H7N9 epidemic?**				
Yes	935	64.48	61.95	66.94
Hard to say	439	30.28	27.93	32.73
No	30	2.07	1.42	2.98
Refused	46	3.17	2.36	4.24

CI = Confidence Intervals.

### Practices

After the emergence of H7N9, 74.41% of participants stated that they had searched for information about H7N9 (Tables 3, 4). This differed by sex, with 79.09% of males and 72.21% of females (P < 0.05) reporting searching for information. The most common method of obtaining information was “use internet” (76.92%), followed by “watch TV” (67.56%), and “read newspaper” (56.26%). Males, younger participants, and those with a higher education level were more likely to choose “use internet” (P < 0.05). However, females and older are more likely to choose “watch TV” and “read newspaper” (P < 0.05). Compared with the middle- or high-income groups, the low-income group had a significantly higher (P < 0.05) proportion of “watch TV” (70.31% vs. 64.72%).

**Table 3:** Precautionary practices against A(H7N9) influenza in Guangzhou, 17–24 June 2013

**Items**	**Total**		**Sex**				**Age**				
	**No.**	**%**	**Male**	**Female**	**χ2**	**P**	**<25**	**25-40**	**>40**	**Trend χ2**	**P**
**Did you recently search some information about H7N9? (response = “yes”)**	1079	74.41	367 (79.09%)	712 (72.21%)	7.58	0.00*	783 (72.77%)	247 (79.17)	49 (79.03%)	5.92	0.06
If yes, what methods did you use to get information?											
Use internet	830	76.92	297 (80.93%)	533 (74.86%)	5.02	0.03*	633 (80.84%)	173 (70.04%)	24 (48.98%)	34.92	0.00*
Watch TV	729	67.56	231 (62.94%)	498 (69.94%)	5.41	0.02*	511 (65.26%)	183 (74.09%)	35 (71.43%)	7.02	0.03*
Read newspaper	607	56.26	179 (48.77%)	428 (60.11%)	12.66	0.00*	429 (54.79%)	151 (61.13%)	34 (69.39%)	6.57	0.01*
Ask friends	227	21.04	94 (25.06%)	133 (18.68%)	7.01	0.01*	176 (22.48%)	47 (19.03%)	4 (8.16%)	6.46	0.04*
Listen to the radio	148	13.72	49 (13.35%)	99 (13.90%)	0.06	0.80	88 (11.24%)	50 (20.24%)	10 (20.41%)	14.80	0.00*
Consult doctor	138	12.79	59 (16.08%)	79 (11.10%)	5.38	0.02*	101 (12.90%)	34 (13.77%)	3 (6.12%)	2.17	0.33
**Have you injected flu vaccine in the past 3 years? (response = “yes”)**	481	33.17	180 (38.79%)	301 (30.53%)	9.72	0.00*	369 (36.80%)	74 (23.72%)	11 (17.74%)	25.64	0.00*
**After emergence of H7N9, what protective measures have you taken?**											
Washed hands more often than usual	1171	80.76	360 (77.59%)	811 (82.25%)	4.42	0.04*	881 (81.88%)	241 (77.24%)	49 (79.03%)	3.47	0.18
Ventilate room more frequency	990	68.27	312 (67.24%)	678 (68.46%)	0.33	0.56	735 (68.31%)	216 (69.23%)	39 (62.90%)	0.96	0.62
Cancelled or postponed social events	707	48.76	193 (41.95%)	514 (52.13%)	14.01	0.00*	520 (48.33%)	161 (51.60%)	26 (41.94%)	2.46	0.33
Bought some drugs for preparation	582	40.14	175 (37.72%)	407 (41.28%)	1.66	0.20*	434 (40.33%)	121 (38.78%)	27 (43.55%)	0.55	0.76
No longer bought poultries to eat	539	37.17	135 (29.09%)	404 (40.97%)	19.06	0.00*	391 (36.34%)	130 (41.67%)	18 (29.03%)	4.78	0.09
Reduced the amount I go into shops	416	28.69	131 (28.23%)	285 (28.90%)	0.07	0.79	312 (29.00%)	87 (27.88%)	17 (27.42%)	0.20	0.91

*P < 0.05.

**Table 4:** Precautionary practices against A(H7N9) influenza in Guangzhou, 17–24 June 2013

**Items**	**Education**				**Income per capita**					**Living area**		
	**Lower than college graduate**	**College graduate and higher**	**χ2**	**p**	**Less than $6,000**	**>$6,000**	**χ2**	**P**	**Suburban or rural**	**Downtown**	**χ2**	**P**
**Did you recently search some information about H7N9? (response = “yes”)**	581 (76.35%)	498 (72.28%)	3.14	0.08	530 (72.80%)	549 (76.40%)	1.99	0.16	411 (73.66%)	663 (75.08%)	0.37	0.54
If yes, what methods did you use to get information?												
Use internet	418 (71.94%)	412 (82.73%)	17.6	0.00*	414 (78.11%)	416 (75.77%)	0.83	0.36	325 (79.08%)	502 (75.72%)	1.62	0.20
Watch TV	398 (68.50%)	331 (66.47%)	0.51	0.48	343 (64.72%)	386 (70.31%)	3.87	0.04*	282 (68.61%)	445 (67.12%)	0.26	0.61
Read newspaper	324 (55.77%)	283 (56.83%)	0.12	0.73	308 (58.11%)	299 (54.46%)	1.46	0.23	227 (55.23%)	377 (56.86%)	0.27	0.60
Ask friends	123 (21.17%)	104 (20.88%)	0.01	0.90	118 (22.26%)	109 (19.85%)	0.94	0.33	90 (21.90%)	136 (20.51%)	0.29	0.59
Listen to the radio	81 (13.94%)	67 (13.45%)	0.05	0.81	68 (12.83%)	80 (14.57%)	0.69	0.41	60 (14.60%)	87 (13.12%)	0.47	0.49
Consult doctor	84 (14.46%)	54 (10.84%)	3.14	0.07	68 (12.83%)	70 (12.75%)	0.00	0.97	59 (14.36%)	77 (11.61%)	1.72	0.19
**Have you injected flu vaccine in the past 3 years? (response = “yes”)**	260 (34.17%)	221 (32.08%)	0.71	0.40	231 (31.73%)	250 (34.63%)	1.37	0.24	203 (36.38%)	276 (31.26%)	4.04	0.04*
**After emergence of H7N9, what protective measures have you taken?**												
Washed hands more often than usual	605 (79.50%)	566 (82.15%)	1.63	0.20	576 (79.12%)	595 (82.41%)	2.52	0.11	448 (80.29%)	717 (81.20%)	0.18	0.67
Ventilate room more frequency	247 (36.01%)	265 (38.46%)	0.93	0.33	485 (66.62%)	505 (69.94%)	1.85	0.17	391 (70.07%)	592 (67.04%)	1.45	0.23
Cancelled or postponed social events	353 (46.39%)	354 (51.38%)	3.60	0.06	369 (50.69%)	338 (46.81%)	2.18	0.14	271 (48.57%)	430 (48.70%)	0.00	0.96
Bought some drugs for preparation	311 (40.87%)	271 (39.33%)	0.35	0.55	301 (41.35%)	281 (38.92%)	0.89	0.35	218 (39.07%)	359 (40.66%)	0.36	0.55
No longer bought poultries to eat	274 (36.01%)	265 (38.46%)	0.93	0.33	253 (34.75%)	286 (39.61%)	3.66	0.06	221 (39.61%)	315 (35.67%)	2.26	0.13
Reduced the amount I go into shops	207 (27.20%)	209 (30.33%)	0.17	0.19	215 (29.53%)	201 (27.84%)	0.51	0.48	162 (29.03%)	251 (28.43%)	0.06	0.80

*P < 0.05.

Approximately one-fifth (21.04%) of participants chose “ask friends”. This differed between sex, with 25.06% of males, and 18.68% of females reporting “inquire friends (P < 0.05). In addition, the proportion of “ask friends” between those under 25 years old, 25–40 years old, and over 40 years old was 22.48%, 19.03%, and 8.16%, respectively. This trend reached statistical significance (Trend χ2 = 6.46, P = 0.04).

A total of 13.72% of participants reported “listen to the radio” to get H7N9 information. This differed among age groups, with 11.24% of those under 25 years, 20.24% of those aged 25–40 years, and 20.41% of those over 40 years old reporting “listen to the radio” (Trend χ2 = 14.80, P = 0.00). Moreover, 12.79% of participants chose “consult doctor”, which was significantly higher in males than in females (16.08% vs. 11.10%).

Regarding the preventive measures, 80.76% of participants reported washing hands more often than usual, which was significantly higher (P < 0.05) in females (82.25%) than in males (77.59%). In addition, a total of 68.27% reported ventilating rooms (by opening windows and doors) more frequently than before. Roughly half of the participants (48.76%) cancelled or postponed their social events, and this was significantly higher in females than in males (52.13% vs. 41.95%). Of particular note, over one-third (37.17%) of participants reported no longer buying chickens, ducks, geese and other poultry, and this was higher in females than in males (40.97% vs. 29.09%) (Tables 3, 4).

**Figure 2:**
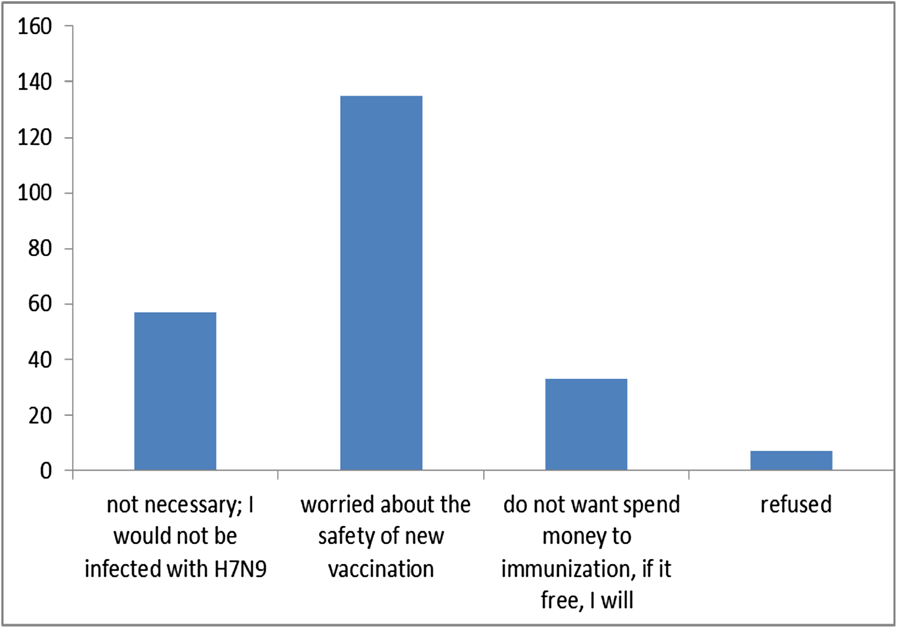
Reasons for participants to accept an A(H7N9) influenza vaccine in Guangzhou, 17–24 June 2013 (N = 232).

Regarding vaccination, 84.00% of participants reported that they would accept an A/H7N9 influenza vaccine if it is available. The primary reason for not accepting the new vaccine was “worry about safety” (58.19%), followed by “not necessary, I would not be infected with H7N9” (24.57%), and “do not want spend money to immunize, if it is free, I will” (14.22%) (Figure 2).

### Information needs

When participants were asked about their information needs if there were an outbreak of H7N9 in Guangzhou, the highest proportion of responses was “how to protect my family from infection?” (75.31%), followed by “what is the current epidemic situation?”(71.86%), “is the vaccination available? Is it safe?” (63.10%), “is there any effective drug treatment?” (62.55%), and “how to conduct home disinfection?” (50.41%) (Table 5).

**Table 5:** Participants’ information needs in response to “once H7N9 outbreaks in Guangzhou”

**Items**	**No.**	**%**	**95% CI lower**	**95% CI upper**
How to protect my family from infection?	1092	75.31	72.99	77.49
What is the current epidemic situation?	1042	71.86	69.46	74.15
Is the vaccination available? Is it safe?	915	63.10	60.55	65.58
Is there any effective drug treatment?	907	62.55	60.00	65.04
How to conduct home disinfection?	731	50.41	47.81	53.02
What kind of food can increase resistance of body to the virus?	713	49.17	46.57	51.78
Can the virus transmit from person to person?	637	43.93	41.36	46.53
How to do if I suspect I am infected with H7N9?	616	42.48	39.93	45.08
How to visit hospital?	589	40.62	38.09	43.20
How can we get reliable information on the disease?	545	37.59	35.10	40.14
What preparatory work has been done by health department?	512	35.31	32.86	37.84

CI = Confidence Intervals.

### Multivariate analysis

Because different methods of obtaining H7N9 information varied significantly with some of demographic variables (indicated by the chi-square test on univariate analysis; Tables 3, 4), we conducted multivariate analyses to determine which independent variables are significant in logistic regression models, with non-significant variables also entered into the models. The results are shown in Tables 6, 7. In model A, sex did not remain significant on multivariate analysis (P > 0.05). Younger (≤40 years) and more education were significantly associated with higher likelihood of using the internet (P < 0.05). Model B indicated that females and those with a higher income had a significantly higher likelihood of watching TV (P < 0.05), and model C indicated that females had a significantly higher likelihood of reading the newspaper (P < 0.05). Model D indicated that being male and younger (under 25 years) was significantly with higher likelihood of inquiring friends (P < 0.05), model E indicated that older participants (over 40 years) were significantly more likely to listen to the radio (P < 0.05), and model F indicated that being male was significantly associated with a higher likelihood of consulting a doctor (P < 0.05). In addition, because sex and age were both significant in model D, we further conducted an interaction analysis. The interaction term (sex*age) was found to not be significant (P > 0.05), so we excluded it from the final model.

**Table 6:** Multivariate regression analysis of the impact of various factors on the methods of obtaining H7N9 information

**Independent variables**	**Model A: Use internet**			**Model B: Watch TV**			**Model C: Read newspaper**		
	**P**	**OR**	**OR 95% CI**	**P**	**OR**	**OR 95% CI**	**P**	**OR**	**OR 95% CI**
Sex									
Female	-	1.00	-	-	1.00	-	-	1.00	-
Male	0.13	1.28	0.92-1.75	0.03*	0.74	0.56-0.96	0.00*	0.63	0.48-0.81
Annual income									
≤$6,000	-	1.00	-	-	1.00	-	-	1.00	-
**>**$6,000	0.19	0.82	0.61-1.10	0.04*	1.29	1.11-1.67	0.17	0.84	0.66-1.07
Education level									
Lower than college graduate	-	1.00	-	-	1.00	-	-	1.00	-
College graduate and higher	0.00*	1.62	1.18-2.19	0.80	0.97	0.74-1.25	0.29	1.15	0.89-1.47
Living area									
Suburban or rural	-	1.00	-	-	1.00	-	-	1.00	-
Downtown	0.65	0.93	0.68-1.26	0.47	0.91	0.69-1.18	0.82	1.03	0.80-1.32
Age									
>40 years	-	1.00	-	-	1.00	-	-	1.00	-
25-40 years	0.02*	2.17	1.15-4.08	0.70	1.15	0.57-2.28	0.37	1.34	0.71-2.49
<25 years	0.00*	3.58	1.95-6.52	0.43	0.77	0.40-1.47	0.94	1.02	0.56-1.85
Constant	0.04*	1.17	-	0.01*	2.60	-	0.08*	1.40	-

CI = Confidence Intervals, OR = Odds Ratio.

*P < 0.05.

**Table 7:** Multivariate regression analysis of the impact of various factors on the methods of obtaining H7N9 information

**Independent variables**	**Model D: Inquire friends**			**Model E: Listen to the radio**			**Model F: Consult doctor**		
	**P**	**OR**	**OR 95% CI**	**P**	**OR**	**OR 95% CI**	**P**	**OR**	**OR 95% CI**
Sex									
Female	-	1.00	-	-	1.00	-	-	1.00	-
Male	0.01*	1.47	1.08-1.99	0.91	0.98	0.67-1.42	0.02*	1.56	1.07-2.25
Annual income									
≤$6,000	-	1.00	-	-	1.00	-	-	1.00	-
**>**$6,000	0.36	0.87	0.64-1.16	0.47	1.14	0.80-1.62	0.90	1.03	0.71-1.47
Education level									
Lower than college graduate	-	1.00	-	-	1.00	-	-	1.00	-
College graduate and higher)	0.44	0.89	0.65-1.20	0.69	1.08	0.74-1.55	0.09	0.77	0.66-1.04
Living area									
Suburban or rural	-	1.00	-	-	1.00	-	-	1.00	-
Downtown	0.91	0.98	0.72-1.33	0.31	0.83	0.58-1.19	0.22	0.79	0.55-1.14
Age									
**>**40 years	-	1.00	-	-	1.00	-	-	1.00	-
25-40 years	0.08	2.64	0.89-7.73	0.86	0.93	0.43-2.01	0.15	2.45	0.71-8.36
<25 years	0.03*	3.24	1.13-9.22	0.04*	0.46	0.21-0.96	0.17	2.34	0.70-7.75
Constant	0.00*	0.09	-	0.00*	0.28	-	0.00*	0.07	-

CI = Confidence Intervals, OR = Odds Ratio.

*P < 0.05.

We also conducted multivariate analysis of willingness to accept H7N9 vaccine, with possible predictor variables and demographics (including sex, age group, marital status, education level, living area, annual income, and place of birth) included in the logistic regression model. Influenza vaccination within the past 3 years (OR = 1.97, P < 0.05) and worry about being infected with the A/H7N9 (compared to “not worried”, OR for “worried” = 2.06, OR for “more worried” = 2.78, and OR for “very worried” = 3.58; P < 0.05 for all three) were significantly associated with willingness to receive an H7N9 vaccine (Table 8).

**Table 8:** Multivariate regression analysis of the impact of various factors on willingness to accept H7N9 vaccine

**Independent variables**	**B**	**SE**	**P**	**OR**	**95% CI for OR**
Sex					
Female	-	-	-	1.00	-
Male	0.10	0.16	0.54	1.11	0.80-1.51
Education level					
Lower than college graduate	-	-	-	1.00	-
College graduate and higher	-0.14	0.15	0.36	0.87	0.64-1.17
Living area					
Suburban or rural	-	-	-	1.00	-
Downtown	0.03	0.15	0.86	1.03	0.76-1.38
Annual income					
≤$6,000	-	-	-	1.00	-
**>**$6,000	0.22	0.15	0.13	1.25	0.93-1.67
Age					
**>**40 years	-	-	-	1.00	-
25-40 years	-0.10	0.36	0.79	0.91	0.44-1.84
<25 years	0.03	0.18	0.85	1.03	0.72-1.47
Place of birth					
Others	-	-	-	1.00	-
Guangzhou	0.04	0.22	0.73	1.04	0.96-1.71
Marital status					
Single	-	-	-	1.00	-
Married/once married	0.11	0.31	0.52	1.11	0.86-1.58
Received influenza vaccine in recent 3 years					
No	-	-	-	1.00	-
Yes	0.68	0.18	0.00*	1.97	1.38-2.78
Worry about being infected with H7N9					
No worry	-	-	-	1.00	-
Not to matter	-0.24	0.34	0.48	0.79	0.40-1.52
Worry	0.73	0.18	0.00*	2.06	1.45-2.94
More worry	1.02	0.23	0.00*	2.78	1.78-4.33
Very worry	1.28	0.26	0.00*	3.58	2.15-5.95
Constant	0.82	0.21	0.00*	-	-

CI = Confidence Intervals, OR = Odds Ratio.

*P < 0.05.

## Discussion

To our knowledge, this is the first study that focuses on understanding the public's awareness of and attitudes toward influenza A (H7N9). In a previous study designed to assess the implications of public understanding of avian influenza, researchers found that the majority of participants did not believe a pandemic would occur, and believed that dealing with the disease was the responsibility of the government [14]. Opinions about the credibility of health information varied from distrust to belief in the credibility of information released by the local health department [11]. Our current study demonstrated that 69.72% of participants worried about being infected with the A/H7N9. Despite the fact that the majority of participants (73.03%) thought the H7N9 information published by the government was accurate and transparent, over one-fifth (20.28%) complained it was not timely enough, and 20.76% thought some information was intentionally concealed by the government. Furthermore, when asked “do you believe that the government can control the H7N9 epidemic?”, nearly one-third of participants (30.28%) responded with “hard to say”. These findings reflect some distrust in the announcements of the health department. This may be because the public did not accept the long interval between the time when cases were identified and the time that information was released to public, as well as the increasing number of cases and infected areas.

We found that after the emergence of H7N9, a majority of participants actively searched for information about H7N9, and the primary method of obtaining information was using the internet. This result is consistent with other studies that indicated that the internet is increasingly used by the public as the most important source of health-related information [15]. Furthermore, our data also indicate that different methods of obtaining information were significantly associated with different demographic variables. For example, younger and more educated participants were significantly more likely to use the internet, while female and higher-income participants were significantly more likely to watch TV. Females were also more likely to read a newspaper for information. Although few participants (13.72%) chose “listen to the radio”, older participants were more likely to get information from the radio than younger participants. This suggests that the transmission of heath information should consider the demographic characteristics of the target audience in determining which methods to emphasize. In addition, it is worth noting that among the young participants, over one-fifth chose “ask friends” rather than seeking a more formal information source. This should be addressed in targeting intervention efforts specifically at young people.

Our study demonstrated that most subjects (80.76%) reported washing their hands more often than usual. Similar findings were also reported at the beginning of the H1N1 influenza pandemic in Hong Kong [16], and in the United Kingdom, 28% of subjects reported changing their hand washing behavior as a result of H1N1 influenza [10]. We also found that of participants 68.27% reported ventilating rooms more frequently than before, and nearly half (48.76%) cancelled or postponed their social events because of A/H7N9. When viewed together, the data from these studies implies that preventative health behaviors become more prevalent during infectious disease epidemics. Furthermore, our data revealed that men were less likely to adopt comprehensive precautionary measures against H7N9, such as washing hands and reducing social events. A deeper understanding of the root causes of such differential risk behavior can help inform the development of dissemination strategies directed at different subgroups. Several previous studies have also indicated that men are less likely to follow behavioral recommendations (such as hand washing) to prevent the transmission of H1N1 influenza, SARS, and other infectious diseases [17-19]. Therefore, men need special targeting for health education, especially to improve their knowledge of influenza, because knowledge of influenza and perceived effectiveness of personal hygiene practices (PHPs) has been shown to be associated with PHPs [20].

We found that more than one-third of participants stated that after the emergence of H7N9, they no longer bought chickens or other poultry to eat. A previous study has revealed that perceived theoretical threat from poultry was associated with less buying of live poultry [21]. We should be aware that the prolonged warning that a future pandemic could be sparked by avian influenza viruses is likely to cause pandemic fatigue in the public, and would probably not change their perception of avian influenza risk and associated protective behavior [22]. Some causal beliefs and lay perceptions of avian influenza contradicted public health efforts at control [23]. Therefore, more effort should be made to improve compliance of proper preventive measures and reduce panic among the public. In addition, because the Guangzhou population faces risks from the high prevalence of exposure during purchase and poultry rearing [24], better management for raising and selling poultry in Guangzhou is needed.

The high proportion (84.00%) of participants indicating a willingness to receive H7N9 vaccine if it is available bodes well for influenza prevention through vaccination in Guangzhou. Of those whose response was “no”, the primary reason for unwillingness to accept a vaccine was concern about the safety of the new vaccine. A similar finding was also observed during the pH1N1 pandemic in Hong Kong, which indicated that perceived risk from the pH1N1 vaccine could inhibit pH1N1 vaccine uptake [25]. These results suggest that some participants lacked an understanding of the process of developing influenza vaccine based on the probability of strains. While we only studied a small subset of the population in Guangzhou, if these results were found to be representative, educational materials distributed about the novel influenza vaccine should focus on its safety record, manufacturing, and the similarities between seasonal influenza vaccination and the H7N9 vaccine. These efforts could help to dispel these fears, considering that we found that participants who had received influenza vaccine within recent three years were nearly two times more likely to accept H7N9 vaccine compared with those who had not.

Although the interviewees responded a relative high willingness to receive H7N9 vaccine, in the past three years, only 33.17% actually received the seasonal influenza vaccine. This implied that the current high willingness of accepting vaccine may be attributed to the high case fatality rate of H7N9 reported, and a high proportion of participants fearing they will be infected. As indicated in this study, worry was found to be the strongest predictor of vaccination uptake. Consistent with our finding, Liao et al. also reported that perceived low risk from pH1N1 could inhibit pH1N1 vaccine uptake [21]. That means if the public believes that the severity of A/H7N9 is lower, the acceptance rate may decline. During the early stage of the pH1N1 pandemic (May-June, 2009), international studies assessing willingness to receive the pH1N1 vaccine indicated rates that ranged from 36.9% [26] to 49.6% [27]. However, national data from Australia collected in November and December 2009, when the public believed that the pandemic was coming to an end [8], showed that there had only been a 14% uptake of the vaccine [28]. Therefore, combining our finding with previous published literatures suggests that when levels of worry are generally low, acting to increase the volume of mass media and advertising coverage is likely to increase the perceived efficacy of recommended behaviors, which, in turn, is likely to increase their vaccination uptake.

We showed that in response to “once H7N9 outbreaks in Guangzhou”, participants’ main concerns included “how to protect my family from infection?”, “what is the current epidemic situation?”, “is the vaccination available? Is it safe?”, “is there any effective drug treatment?”, and “how to conduct home disinfection?”. This is similar to Aihua et al., who reported that during the 2009 H1N1 influenza pandemic, the public's primary concern was effective and easy-to-operate preventive measures [29]. Therefore, these information needs should be taken into account in future health education campaigns.

Some limitations of this study must be acknowledged. First, our subjects were employees of food production and operation, and we recognize the limitations of applying the results of this study to the general population. Second, this survey measured the participants’ views at a specific point in time; therefore, the attitudes and practices reflected the information available at that time. Third, some of the questions in our questionnaire had Likert-type response options, which restricted the preferences of participants to a few options. A fourth limitation is inherent to the study design: the use of convenience sampling - as opposed to random sampling - imposes some inherent selection bias and diminishes the internal validity.

## Conclusions

Taken together, despite these limitations, our study provides valuable insight into attitudes and practices related to H7N9 influenza among employees of food production and operation just three months after the first human infection case reported in China, and one month after chickens were found to be infected in Guangzhou. We found that: 1) The majority of participants worried about being infected with the A/H7N9 and took initiative to find information about H7N9. The internet, television, and newspapers were the main methods of obtaining information, and the methods differed by sex, age, and some other demographic variables. 2) Quite a number (20.28%)of participants complained that the information was not timely enough, and 20.76% believed information was intentionally concealed by the government. Nearly one-third of participants did not firmly believe that the government could control the H7N9 epidemic. These results reflect some distrust in the health department. 3) Most participants took positive measures to prevent infection; however, more than one-third reported no longer buying chickens and other poultry to eat. A majority of participants indicated a willingness to receive an A/H7N9 vaccine, and the primary reason for unwillingness to receive a vaccine was concern about safety. A history of influenza vaccination and worry about being infected with the A/H7N9 were significantly associated with the intention to receive an H7N9 vaccine. This suggests that more effort should be made to improve compliance of proper preventive measures and reduce panic among the public. In addition, we also reported the public's main information needs if a human H7N9 outbreak occurs in Guangzhou. These findings should be used to improve health education and develop a correct strategy for H7N9 control and prevention.

### Consent

Written informed consent was obtained from the patient for the publication of this report.

## Abbreviations

SARS: Severe acute respiratory syndrome; UK: United Kingdom; CDC: U.S. center for disease control and prevention; TV: Television; pH1N1: Pandemic (H1N1) 2009.

## Competing interests

The authors declare that they have no competing interests.

## Authors’ contributions

All authors contributed to the design and execution of the study and analyses. TGL contributed to the conception of study and interpretation and writing of the manuscript. JF participated in the conception of the study and drafting of the manuscript. PZQ participated in the design and data collection. WSL participated in the design and statistical analysis. MXL participated in the design and data collection. XMF contributed to the manuscript writing. MW contributed to the study design, interpretation, and manuscript writing. All authors read and approved the final manuscript.

## Pre-publication history

The pre-publication history for this paper can be accessed here:

http://www.biomedcentral.com/1471-2334/14/4/prepub

## Supplementary Material

Additional file 1 Questionnaire on investigation of attitudes, practices and information needs regarding novel influenza A (H7N9). Click here for file
