# Supplementary material for: Attitudes, practices and information needs regarding novel influenza A (H7N9) among employees of food production and operation in Guangzhou, Southern China: a cross-sectional study
Source: BMC Infect Dis. 2014 Jan 2;14:4. doi: 10.1186/1471-2334-14-4 (PMC3899619; doi:10.1186/1471-2334-14-4)
Supplement: Additional file 1 — Questionnaire on investigation of attitudes, practices and information needs regarding novel influenza A (H7N9). [file 1471-2334-14-4-S1.doc]

**Form ID：**

**Team number:**

**Date:**

**Q1. Did you recently search some information about H7N9?**

- Yes
- No

**Q1a. If yes, what methods did you used to get information?**

- Search from web
- Watch news from TV
- Read news from newspaper
- Inquire from friends
- Listen to the radio
- Consult form doctor

**Q2. Are you worried about being infected with H7N9?**

- Very worried
- More worried
- Worried
- Not to matter
- No worried
- Absolutely not worried

**Q3.Have you injected flu vaccine in the past three years?**

- Yes
- No

**Q4. If the H7N9 vaccination is available, would you like to accept injection?”.**

- Yes
- No

**Q4a. If no, why?**

- Worry about safety of new vaccination
- Not necessary; I would not be infected with H7N9
- Do not want spend money to immunization, if it is free, I will

**Q5. After emergence of H7N9, what protective measures have you taken?**

- Washed hands more often than usual
- Increased ventilation frequency of bedroom
- Cancelled or postponed social events
- Initiative to buy the drugs for preparation
- No longer bought poultries to eat
- Reduced the amount I go into shops

**Q6. How do you think about the H7N9 information published by government during this event?**

- Accurate and transparent
- Not timely enough
- Difficult to understand more puzzling
- Publics have limited access to get correct and authority information
- The information was intentionally concealed by Government
- The severity of epidemic was deliberately exaggerated by Government, causing panic

**Q7. How about your personal satisfaction evaluation on the measures taken by government presently?**

- Very dissatisfied
- Dissatisfied
- Satisfied
- More satisfied
- Very satisfied

**Q8. Do you believe that government can control the H7N9 epidemic?**

- Yes
- Hard to say
- No
- Refused

**Q9. Once H7N9 outbreaks in Guangzhou”, what are your main concerns?**

- How to protect my family from being infected with this virus?
- What is the current epidemic situation?
- Is the vaccination available? Is it safe?
- Is there any effective drug treatment?
- How to conduct home disinfection?
- What kind of food can increase resistance of body to the virus?
- Can the virus transmit from person to person?
- How to do if I suspect I am infected with H7N9?
- How to visit hospital?
- How can we get reliable information on the disease?
- What preparatory work has been done by health department?
- Else

**Q10. What is your age?** （ ）years old

**Q11. What is you current marital status?.** 🗆 Single 🗆 Married/once married

**Q12. Where is you birth place?** 🗆 Guangzhou 🗆 Others

**Q13. What is your highest education level completed?**

- Illiterate
- Elementary school graduate
- Middle school graduate
- High school graduate
- 1-3 years or technical school training
- College graduate
- Graduate or professional school

**Q14. What is your annual household income per capita?**

- Less than $2,000
- $2,001-$6,000
- $6,001-$10,000
- $10,001-$20,000
- >$20,000

**Q15. You sex：**

- male
- female

**Q16. What is your living area?**

- Xuexiu District
- Liwan District
- Huangpu District
- Haizhu District
- Tianhe District
- Baiyun District
- Huadu District
- Conghua District
- Zengcheng District
- Nansha District
- Luogang District
- Panyu District

**Team leader check and sign name**
